# Supplementary material for: Measuring social mobility in temporal networks
Source: Sci Rep. 2025 Feb 18;15:5941. doi: 10.1038/s41598-025-89090-1 (PMC11836308; doi:10.1038/s41598-025-89090-1)
Supplement: Supplementary file 1 — Supplementary Information. [file 41598_2025_89090_MOESM1_ESM.pdf]

Supplementary Information for Measuring social mobility in temporal networks

Matthew Russell Barnes, Vincenzo Nicosia, Richard G. Clegg

January 3, 2025

1 Data Corpus Details

There are many different varieties of systems which can be modelled using temporal networks. Therefore, we have gathered a corpus of 26 network datasets taken from real world systems. The networks vary in size from just over 100 edges to more than one million and all networks are treated as undirected and unweighted with added their links never being removed. All of the networks can be seen in Table S1 with a description for each.

We have assigned each dataset a "collection type" based on where the data was collected. Social data is gathered from a social media site, email list or messaging app; nodes are people and edges are emails, messages or "follows" between two people. Co-occurrence data is gathered from books, articles and musical notation; nodes are words or musical notes and the edges are created when two nodes exist next to one another in the source material. Citation data is gathered from literature which cites other literature; nodes are pieces of literature and edges are the citations. Economic data is gathered from places where financial transactions are of interest; nodes are products, buyers and sellers and edges are linking buyers/sellers with their products. Computer data is gathered from computer networks; nodes are computers and edges are interactions between the computers. Transport data is gathered from transportation networks; nodes are geographical places and edges are the transportation links between the places. Finally, contact data is gathered when people interact in close proximity, usually using proximity sensors; nodes are people and edges are created when two people are physically close for a threshold of time.

We also assign a "link creation type" based on the dynamics of the networks themselves. Stars consist of all edges connecting to one node, for example when one paper cites others. Individual edges are created when only two nodes interact, for example two characters in a book. Cliques are fully interconnected node clusters, for example everyone who answers a question on StackOverflow is connected together. Bipartite networks consist of two separate node pools which only link with each other, and not within themselves. Finally, spatial networks are a mix of many of the others and so have been separated into their own category.

For each network, we treat each edge event as one time iteration for the network, where all edges that happen simultaneously are grouped into the same edge event. The whole time period is split into five equal time windows, this is to ensure any long periods of inactivity in the network do not leave empty spaces in our plots. Then, we focus on the first window and divide it into two windows of equal length. We build a network snapshot for both of these two sub-windows, and calculate the mobility taxonomy correlations between them. Then, we push the time window forward by $\frac{1}{2}$ of its duration. This is repeated until the time window reaches the end of the whole time period of the data. Therefore, we calculate 9 correlations for each network, each taking into account $\frac{1}{5}$ of the total time period of the network.

In Figure S1, we plot each network in the corpus individually. This plot is much harder to interpret than Fig. 1 which uses standard deviations to give insight into the ranges of correlation values for each network collection type.

	Name	Description	Collection	Link Creation	Nodes	Edges
a	College Messages [11]	Messages between students on a UC-Irvine message board	Social	Star	1,899	59,835
b	SCOTUS Majority [4]	Legal citations among majority opinions by SCOTUS	Citation	Star	34,613	202,167
c	Amazon Ratings [10]	Amazon user connects to all products they have rated	Economic	Bipartite	3,376,972	5,838,041
d	Citation US Patents [6]	Citation among patents in the United States	Citation	Star	3,774,362	16,516,270
e	Classical Piano [13]	Transitions of chords in western classical piano music	Co-occurrence	Individual	144,183	585,154
f	Email EU [12]	E-mails between users at an EU research institution	Social	Star	986	332,334
g	Procurement EU [17]	Public EU procurement contracts	Economic	Bipartite	839,824	4,098,711
h	Facebook Wall [16]	Posts by users on other users' Facebook wall	Social	Star	46,952	876,993
i	Lord Of the Rings [2]	Character co-occurrence in Lord of the Rings Trilogy	Co-occurrence	Individual	139	2,649
j	PhD Exchange [14]	Exchange of PhD mathematicians between unis in the US	Citation	Star	230	9,584
k	Programming Languages [15]	Influence relationships among programming languages	Citation	Star	366	762
l	Reuters Terror News [3]	Word co-use in Reuters 9/11 coverage	Co-occurrence	Individual	13,308	148,035
m	Route Views [2]	Route Views internet topology	Computer	Individual	33,804	94,993
n	Reddit Hyperlinks Body [9]	Subreddit-to-subreddit hyperlinks from body of posts	Social	Clique	35,776	286,561
o	Reddit Hyperlinks Title [9]	Subreddit-to-subreddit hyperlinks from title of posts	Social	Individual	54,075	571,927
p	Hypertext Conference [7]	Contacts among attendees of ACM Hypertext 2009	Contact	Spatial	113	20,818
q	Infectious [7]	Contacts during Infectious SocioPatterns 2011 event	Contact	Spatial	10,972	415,912
r	Office [5]	Contacts between individuals in an office building	Contact	Spatial	92	9,827
s	AskUbuntu [12]	User answers or comments on questions on AskUbuntu	Social	Clique	159,316	964,437
t	MathOverflow [12]	User answers or comments on questions on MathOverflow	Social	Clique	24,818	506,550
u	StackOverflow [12]	User answers or comments on questions on StackOverflow	Social	Clique	2,601,977	63,497,050
v	SuperUser [12]	User answers or comments on questions on SuperUser	Social	Clique	194,085	1,443,339
x	UCLA AS [2]	UCLA AS level internet topology	Computer	Individual	38,219	226,729
y	US Air Traffic [12]	Flights among all commercial airports in the US	Transport	Individual	2,278	6,390,340
z	Wiki Talk [12]	Wikipedia users editing each other's Talk page	Social	Individual	1,140,149	7,833,140
A	IETF [8]	IETF mailing list replies	Social	Clique	23,792	989,740

Table 1: Dataset corpus used for network creation.

However, this plot clearly shows the two low *mobility* and *neighbour mobility* networks which are referred to in the main paper. The first of these is Citation US Patents [6] which has unusually high *philanthropy* correlation suggesting patents citing highly cited patents themselves receive a large amount of patents. The other is Infectious [7] which has a recurring "jump" in each correlation, this is due to a sudden large decrease in the amount of active nodes in the network (the authors have not managed to verify the cause of this decrease). Finally, the large shift in the Lord Of the Rings [2] philanthropy and *community* correlations, as referred to in the main paper, can be seen more easily in this plot.

2 Extra artificial data results

Table 2 describes the twelve models used in the artificial data study. The models chosen were taken from the literature combined with models that the authors reasoned might maximise or minimise the taxonomy statistics being investigated. As mentioned in the main text, each model uses the basic structure of the classic preferential attachment model [1] where one node is introduced at every iteration and three links. The three links are connected to an existing node with probabilities chosen according to certain rules, for example the classic preferential attachment rule is that the probability of connecting to a node is precisely proportional to its degree.

The figures 2 and 3 show precisely which models were used in the maximisation and minimisation experiments. Each of the ten runs for each experiment is a separate row. For maximisation we can see that for maximising neighbour mobility and mobility the strategy was almost always philanthropy with occasionally the preferential attachment strategy. It makes sense that the strategy that maximises the correlation between degree in one time slice and the next does the same for a neighbourhood. For maximising philanthropy the sum neighbour degree was usually the most

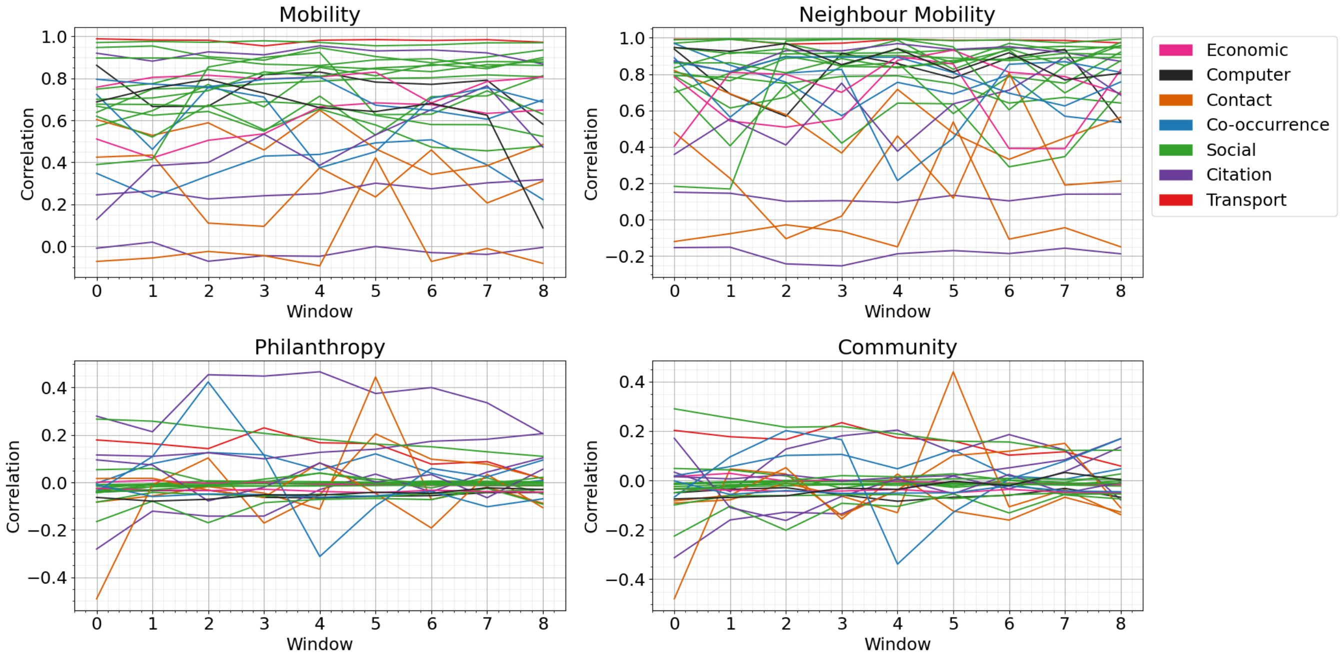

Figure 1: All networks' mobility taxonomy correlations in the corpus coloured by data type. The x-axis represents the ordered window numbers starting at the first timestamp of the network. The y-axis shows the correlation coefficient as measured using Pearson correlation.

Name	Probability of Receiving Edge
Random	Each node is equal
Preferential attachment	Proportional to each node's individual degree
Preferential neighbour attachment	First, a node is chosen using preferential attachment (PA); then one of its neighbours is chosen using PA
Equality	Inversely proportional to each node's individual degree
Sum neighbour degree	Proportional to the sum of the degree of the neighbours of each node
Average neighbour degree	Proportional to the average of the degree of the neighbours of each node
Inverse average neighbour degree	Inversely proportional to the average of the degree of the neighbours of each node
Cluster	Proportional to the clustering coefficient of each node
Eigen	Proportional to the eigenvector centrality of each node
Fitness	Proportional to a sample taken from a $\Gamma(x)$ distribution
Gamma	Same as above but every node is re-sampled every m iterations
Gamma individual	Same as above but only one node is re-sampled

Table 2: Models used for generating the evolutionary dynamics of artificial networks.

successful with occasionally eigen. Finally maximising community took an alternating strategy with average neighbour degree alternating with eigen and sum neighbour degree.

For minimisation equality and sometimes inverse average neighbour degree minimised philanthropy and community. These two strategies would tend to produce networks where all nodes have a similar degree. Minimising neighbour mobility and mobility itself both took an alternating strategy with a complex mix of strategies involved.

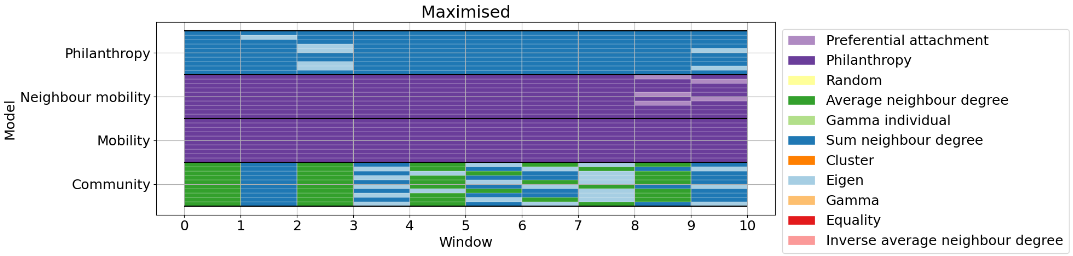

Figure 2: Visualisation of the evolution models used in the maximisation of mobility taxonomy aspects. The x-axis represents the number of 1,000 iteration-long windows (not including the initialisation of the graph).

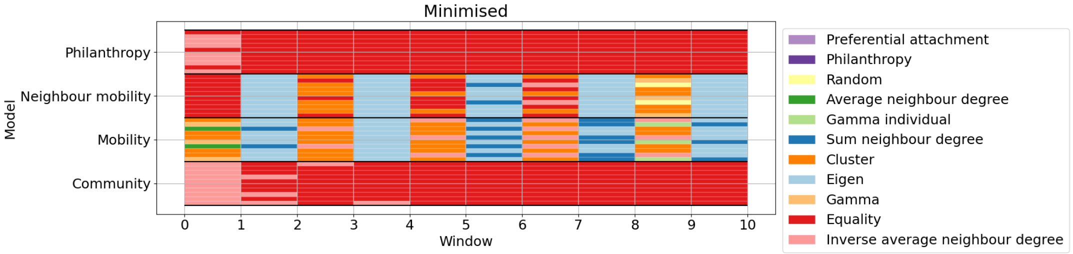

Figure 3: Visualisation of the evolution models used in the minimisation of mobility taxonomy aspects. The x-axis represents the number of 1,000 iteration-long windows (not including the initialisation of the graph).

References

[1] Albert László Barabási and Réka Albert. "Emergence of scaling in random networks". In: *Science* 286.5439 (Oct. 1999), pp. 509–512. ISSN: 00368075. DOI: 10.1126/science.286.5439.509.

[2] Richard G. Clegg et al. "Measuring the Likelihood of Models for Network Evolution". In: *Proc. INFOCOM*. 2009, pp. 272–277. DOI: 10.1109/INFCOMW.2009.5072162.

[3] Steven R Corman, Timothy Kuhn, and Robert D Mcphee. "Studying Complex Discursive Systems". In: *Human Communication Research* (2002). DOI: 10.1111/j.1468-2958.2002.tb00802.x.

[4] James H. Fowler and et al. "Network analysis and the law". In: *Political Analysis* 15.3 (2007), pp. 324–346. ISSN: 10471987. DOI: 10.1093/pan/mpm011.

[5] Mathieu Génois and et al. "Data on face-to-face contacts in an office building suggest a low-cost vaccination strategy based on community linkers". In: *Network Science* 3.3 (2015), pp. 326–347. ISSN: 20501250. DOI: 10.1017/nws.2015.10.

[6] Bronwyn H. Hall and et al. "The NBER patent citation data file: Lessons, insights and methodological tools". In: *NBER* (2001). ISSN: 17762588. DOI: 10.3386/w8498.

[7] Lorenzo Isella and et al. "What's in a crowd? Analysis of face-to-face behavioral networks". In: *JTB* 271.1 (2011), pp. 166–180. ISSN: 00225193. DOI: 10.1016/j.jtbi.2010.11.033.

[8] Prashant Khare et al. "The Web We Weave: Untangling the Social Graph of the IETF". In: *Proceedings of the 16th AAAI Conference on Web and Social Media*. 2022.

[9] Srijan Kumar and et al. "Community Interaction and Conflict on the Web". In: *WWW* (2018), pp. 933–943. DOI: 10.1145/3178876.3186141.

[10] Ee-Peng Lim et al. "Detecting product review spammers using rating behaviors". In: *Proc. CIKM.* 2010, pp. 939–948. DOI: 10.1145/1871437.1871557.

[11] Pietro Panzarasa et al. "Patterns and dynamics of users' behavior and interaction: Network analysis of an online community". In: *Journal of the American Society for Information Science and Technology* 60.5 (2009), pp. 911–932.

[12] Ashwin Paranjape and et al. "Motifs in temporal networks". In: *WSDM, Proc.* (2017), pp. 601–610. DOI: 10.1145/3018661.3018731.

[13] Doheum Park and et al. "Novelty and influence of creative works, and quantifying patterns of advances based on probabilistic references networks". In: *EPJ Data Science* 9.1 (2020). ISSN: 21931127. DOI: 10.1140/epjds/s13688-019-0214-8.

[14] Dane Taylor and et al. "Eigenvector-based centrality measures for temporal networks". In: *MMS* 15.1 (2017), pp. 537–574. ISSN: 15403467. DOI: 10.1137/16M1066142.

[15] Sergi Valverde and Ricard V. Sole. "Punctuated equilibrium in the large-scale evolution of programming languages". In: *JRSI* 12.107 (2015). ISSN: 17425662. DOI: 10.1098/rsif.2015.0249.

[16] Bimal Viswanath et al. "On the evolution of user interaction in facebook". In: *Proceedings of the 2nd ACM workshop on Online social networks.* 2009, pp. 37–42.

[17] Johannes Wachs and et al. "Corruption risk in contracting markets". In: *IJDSA* 12.1 (2021), pp. 45–60. ISSN: 23644168. DOI: 10.1007/s41060-019-00204-1.
